# The phosphoinositide-3 kinase (PI3K)-δ,γ inhibitor, duvelisib shows preclinical synergy with multiple targeted therapies in hematologic malignancies

**DOI:** 10.1371/journal.pone.0200725

**Published:** 2018-08-01

**Authors:** Kerrie Faia, Kerry White, Erin Murphy, Jennifer Proctor, Melissa Pink, Nicole Kosmider, Karen McGovern, Jeffery Kutok

**Affiliations:** Infinity Pharmaceuticals, Inc., Cambridge, Massachusetts, United States of America; University of PECS Medical School, HUNGARY

## Abstract

Duvelisib is an orally active dual inhibitor of PI3K-δ and PI3K-γ in clinical development in hematologic malignancies (HM). To identify novel pairings for duvelisib in HM, it was evaluated alone and in combination with 35 compounds comprising a diverse panel of standard-of-care agents and emerging drugs in development for HM. These compounds were tested in 20 cell lines including diffuse large B-cell, follicular, T-cell, and mantle cell lymphomas, and multiple myeloma. Single agent activity was seen in fourteen cell lines, with a median GI_50_ of 0.59 μM. A scalar measure of the strength of synergistic drug interactions revealed a synergy hit rate of 19.3% across the matrix of drug combinations and cell lines. Synergy with duvelisib was prominent in lymphoma lines with approved and emerging drugs used to treat HM, including dexamethasone, ibrutinib, and the BCL-2 inhibitor venetoclax. Western blotting revealed that certain duvelisib-treated cell lines showed inhibition of phosphorylated (p) AKT at serine 473 only out to 12 hours, with mTORC2 dependent re-phosphorylation of pAKT evident at 24 hours. Combination with dexamethasone or ibrutinib, however, prevented this reactivation leading to durable inhibition of pAKT. The combination treatments also inhibited downstream signaling effectors pPRAS40 and pS6. The combination of duvelisib with dexamethasone also significantly reduced p-4EBP1, which controls cap dependent translation initiation, leading to decreased levels of c-MYC 6 hours after treatment. In support of the in vitro studies, in vivo xenograft studies revealed that duvelisib in combination with the mTOR inhibitor everolimus led to greater tumor growth inhibition compared to single agent administration. These data provide a rationale for exploring multiple combinations in the clinic and suggest that suppression of mTOR-driven survival signaling may be one important mechanism for combination synergy.

## Introduction

Phosphoinositide-3 kinases (PI3Ks) are members of a unique and conserved family of intracellular lipid kinases that phosphorylate the 3’-OH group on phosphatidylinositols or phosphoinositides. The class I PI3Ks (p110α, p110β, p110δ, and p110γ) are typically activated by tyrosine kinases or G-protein coupled receptors to generate phosphatidylinositol (3,4,5)-trisphosphate (PIP3), which engages downstream effectors such as those in the AKT/PDK1 pathway, mTOR, the Tec family kinases, and the Rho family GTPases [1–6]. PI3K-δ and PI3K-γ are preferentially expressed in leukocytes and are important in normal leukocyte function. PI3K-δ and PI3K-γ are proximal in the signal transduction cascades utilized by the adaptive and innate immune system, including B-cell and T-cell receptors, growth factor receptors, and chemokine and cytokine receptor pathways [7,8] and these isoforms also contribute to the development and maintenance of hematologic malignancies [2,3,6, 9–15]. To investigate the hypothesis that simultaneous inhibition of these isoforms would demonstrate anti-tumor growth effects in hematologic malignancies, we developed duvelisib (IPI-145), an orally active, potent dual inhibitor of both PI3K-δ and PI3K- [16]. In clinical trials, duvelisib has shown single agent activity in patients with relapsed/refractory chronic lymphocytic leukemia and indolent non-Hodgkin lymphoma, but was somewhat less active in aggressive lymphomas, such as diffuse large B cell lymphoma (DLBCL) [17–19]. To gain mechanistic insights into the cellular response to duvelisib and identify novel pairings for duvelisib in hematologic malignancies, a high-throughput combination screen in malignant lymphoid cell lines was conducted with a variety of standard-of-care and experimental agents important in lymphoma therapy. Clinically relevant agents were identified that synergized with duvelisib to inhibit growth in DLBCL and transformed follicular lymphoma cell lines, and in vitro analysis revealed a potential mechanism by which these tumor types establish resistance to duvelisib monotherapy through activation of the mTOR pathway.

## Results

### Growth inhibitory activity of duvelisib as single agent

To determine the direct effect of duvelisib on malignant hematologic cells, the growth inhibitory activity of duvelisib was evaluated across a panel of 20 cell lines including those derived from diffuse large B-cell lymphoma, transformed follicular lymphoma, mantle cell lymphoma, multiple myeloma, and T-cell lymphoma. Cell viability was assessed by quantitation of ATP (indicative of a metabolically active cell) after a 72-hour treatment with varying concentrations of duvelisib and reported as growth inhibition (GI). Maximal GI values between 0% and 100% are indicative of a cytostatic effect on growth for duvelisib, whereas, values greater than 100% are indicative of a cytotoxic effect of the drug.

**Fig 1:**
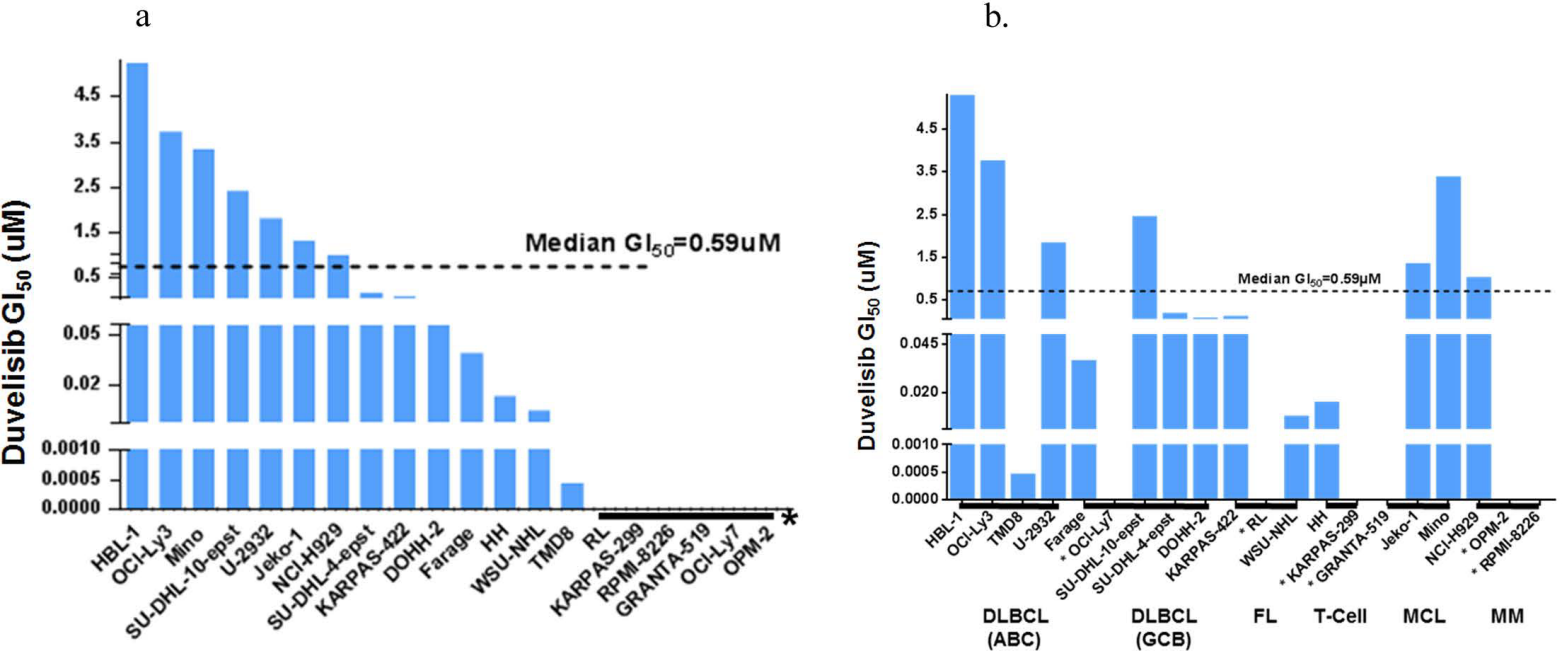
**A. Growth inhibition (GI_50_) of duvelisib across the panel of twenty cell lines**. The median GI_50_ across the cell line panel is 0.59 μM. In six cell lines (**A & B**) with the asterisks (RL, KARPAS-299, RPMI-8226, GRANTA-519, OCI-Ly7, OPM-2), the GI_50_ failed to reach growth Inhibition levels of greater than fifty percent. **B. Growth inhibition (GI_50_) of duvelisib across the panel of twenty cell lines grouped by tumor subtype**. The median GI_50_ across the cell line panel is 0.59 μM. Cell lines are grouped according to tumor subtypes: Diffuse large B cell lymphoma (DLBCL) activated B cell (ABC) and germinal center B cell (GCB), follicular lymphoma (FL), T cell lymphoma (T cell), mantle cell lymphoma (MCL) and multiple myeloma (MM).

Duvelisib was active in 14 out of 20 cell lines with maximal response values greater than 100% in 7 cell lines (Fig 1A and Table 1). The GI_50_ values for duvelisib across this cell line panel are also reported in Table 1, and display a range of sensitivities across a broad array of tumor subtypes. For cell lines where the GI_50_ reached GI levels of greater than fifty percent, the median GI_50_ was 0.59 μM (Fig 1A). In six cell lines (RL, KARPAS-299, RPMI-8226, GRANTA-519, OCI-Ly7, OPM-2), the GI_50_ failed to reach levels of greater than fifty percent (Fig 1B). Several cell lines display considerable sensitivity to duvelisib (GI_50_ values ranging from 0.5 to 200 nM), however, many are weakly sensitive in this experimental setting. Overall, these findings are consistent with direct growth inhibitory effects of duvelisib in a range of lymphoid tumor cell types.

**Table 1:** Activity of duvelisib in a panel of malignant hematologic cell lines.

Cell line	Tumor Type	Maximum Observed Response (% GI)	GI_50_ (μM)
HBL-1	DLBCL (ABC)	58	5.3
OCI-Ly3	DLBCL (ABC)	77	3.7
TMD-8	DLBCL (ABC)	197	0.0005
U-2932	DLBCL (ABC)	75	1.8
Farage	DLBCL (GCB)	148	0.04
OCI-Ly7	DLBCL (GCB)	27	ND
RL	DLBCL (GCB)	30	ND
SU-DHL-10	DLBCL (GCB)	79	2.4
SU-DHL-4	DLBCL (GCB)	169	0.2
Karpas-422	Transformed FL	93	0.1
DOHH-2	Transformed FL	191	0.05
WSU-NHL	Transformed FL	169	0.008
GRANTA-519	Mantle Cell	47	ND
Jeko-1	Mantle Cell	86	1.3
Mino	Mantle Cell	108	3.4
NCI-H929	Multiple myeloma	156	1.0
OPM-2	Multiple myeloma	14	ND
RPMI-8226	Multiple myeloma	25	ND
HH	T-Cell lymphoma	80	0.01
KARPAS-299	T-Cell lymphoma	9	ND

ABC = Activated B cell type; GCB = Germinal B cell type; ND = not determined (%GI < 50)

### Growth inhibitory activity of duvelisib combined with other agents

**Fig 2:**
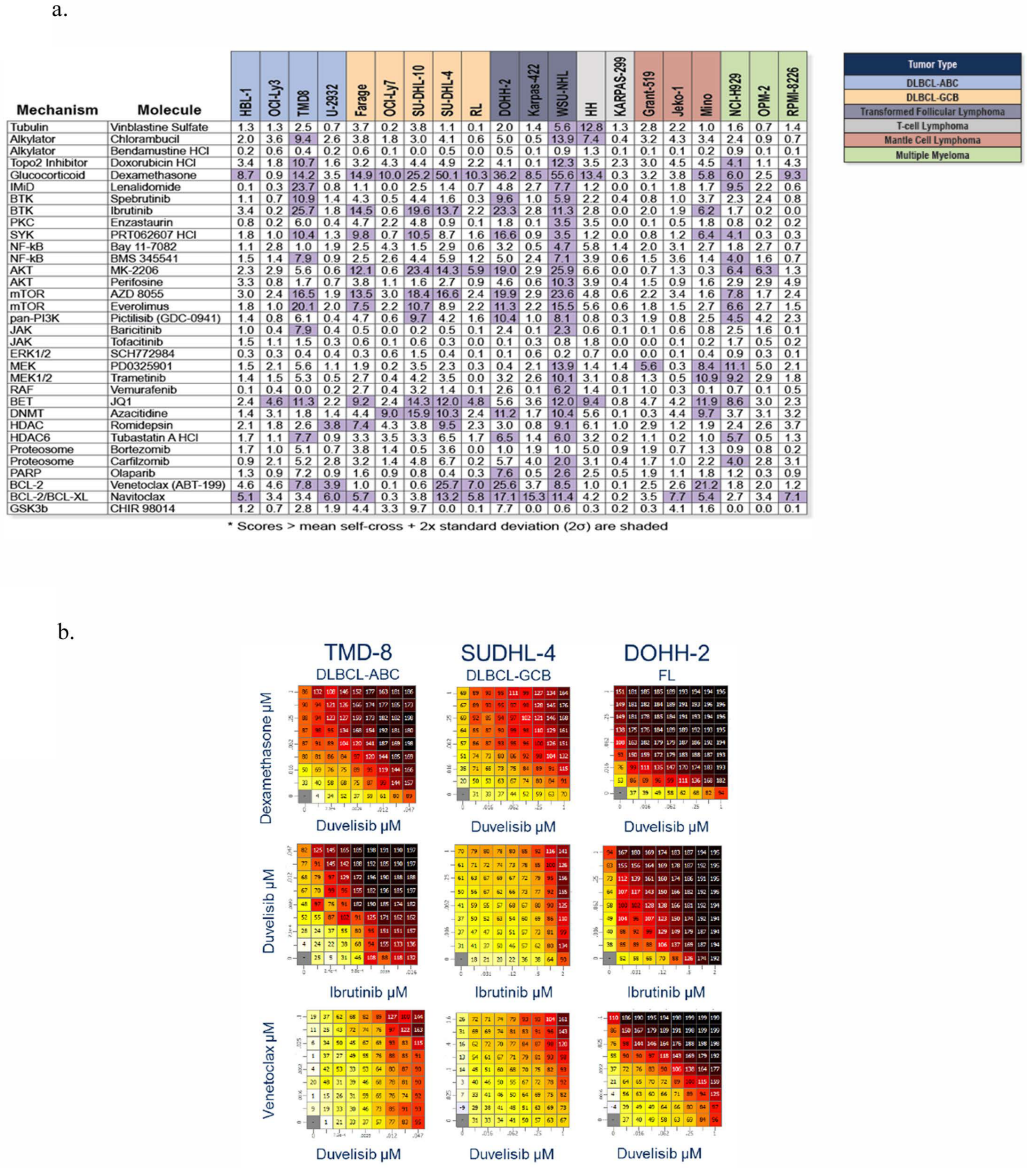
**A. Synergy scores for combination of duvelisib with drugs with different mechanisms of action**. Synergy Scores that exceed the mean self-cross plus two times the standard deviation are highlighted in purple. **B. Synergy score heat maps for duvelisib combined with dexamethasone, ibrutinib and venetoclax.** Representative heat maps for indicated combinations in the TMD-8, SUDHL-4 and DOHH2 cell lines. Scores up to 100 indicate growth inhibition while values >100 indicate cell death.

Duvelisib was evaluated in combination with 35 compounds comprising a diverse panel of standard-of-care agents and emerging drugs in development for hematologic malignancies using a specialized screen (Horizon Discovery) designed to evaluate compound synergy (Fig 2A). To compare to single agent growth inhibition, the same 20 cell line panel was used to evaluate combination effects. The screen measured combination effects in excess of Loewe additivity, and a scalar measure of the strength of synergistic drug interactions termed the synergy score was derived. Filtering on synergy scores exceeding the mean self-cross plus twice the standard deviation revealed a synergy hit rate of 19.3% across the matrix of drug combinations and cell lines (Fig 2A). Interestingly, some cell lines (e.g. WSU-NHL) demonstrated synergy between duvelisib and nearly all agents, while, certain agents (e.g. dexamethasone) combined with duvelisib showed synergy across most cell lines (Fig 2A). Synergy was most prominent in DLBCL and follicular lymphoma lines and was seen with both approved and emerging drugs used to treat B-cell malignancies, including dexamethasone, inhibitors of the B-cell receptor signaling pathway, such as ibrutinib, and the BCL-2 inhibitor venetoclax (Fig 2B).

### Re-phosphorylation of AKT after PI3K inhibition by duvelisib is suppressed by combination with dexamethasone or ibrutinib

**Fig 3:**
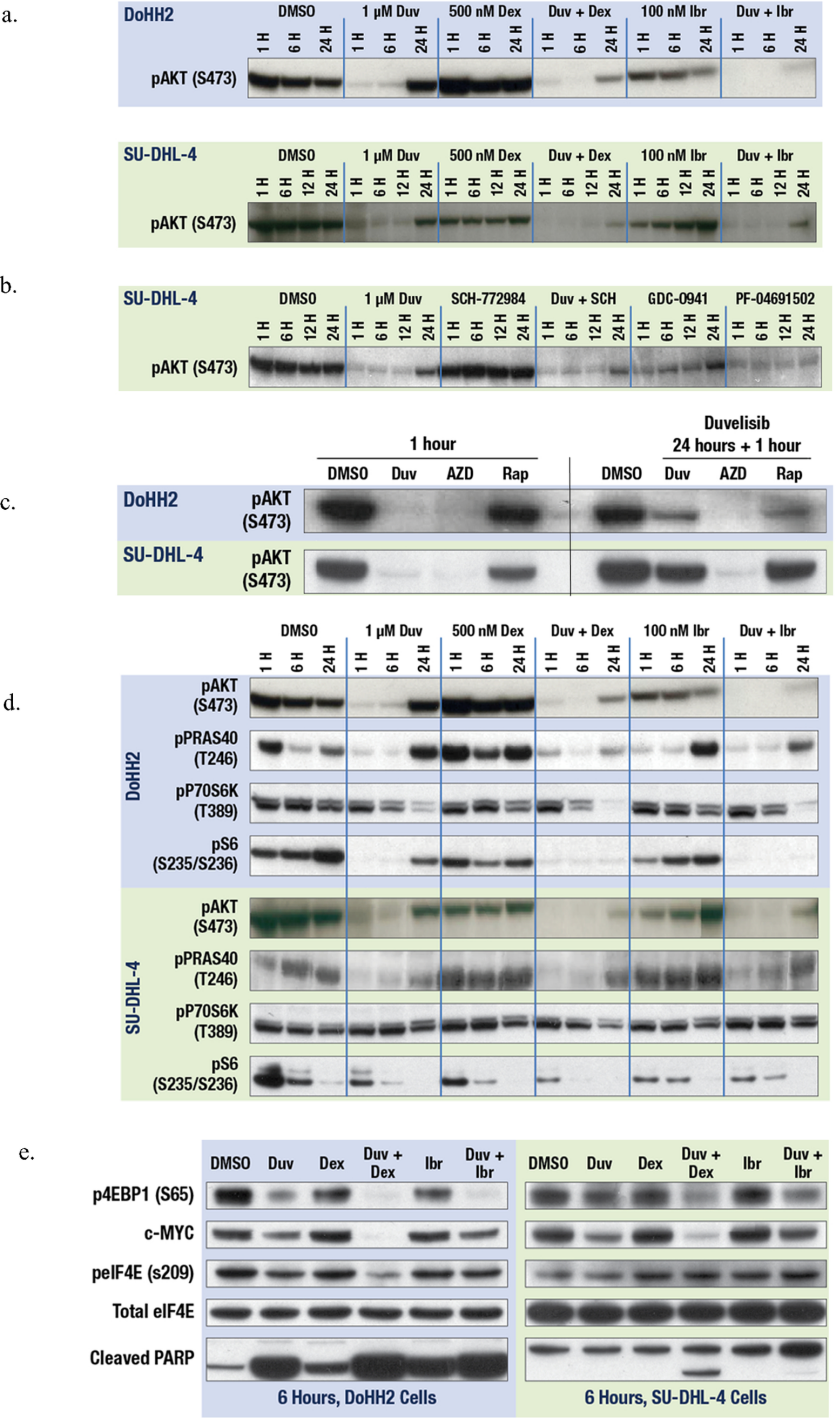
**A Combination treatment of duvelisib plus dexamethasone or ibrutinib leads to sustained inhibition of pAKT**. Western blot analysis of DOHH2 (top) and SU-DHL-4 (bottom) treated with duvelisib (1 μM), dexamethasone (500 nM), ibrutinib (100nM) or the combination of duvelisib plus dexamethasone or ibrutinib for 6 hours or 24 hours and stained with pAKT (S473). **B. mTOR inhibition can suppress re-phosphorylation of pAKT.** SU-DHL-4 cells treated with duvelisib (1 μM), the pan-PI3K inhibitor GDC-0941(500 nM) or the ERK inhibitor SCH-772984 (500 nM) resulted in re-phosphorylation of pAKT (S473) at 24 hours, while combination with the PI3K/mTOR inhibitor PF-04691502 (500 nM) prevented re-phosphorylation of pAKT at 24 hours. **C. Re-phosphorylation of pAKT after duvelisib treatment is dependent on mTORC2**. DOHH2 (top) or SU-DHL-4 (bottom) treated with duvelisib (1 μM), mTOR1/2 inhibitor AZD-8055 (200 nM) or the mTORC1 inhibitor rapamycin (100 nM) for 1 hour (left) or following a 24 hour treatment with duvelisib (1 μM)(right). **D. Duvelisib combinations with dexamethasone and ibrutinib reduce activation of downstream effectors.** DOHH2 (top) and SU-DHL-4 (bottom) were treated with duvelisib, dexamethasone and ibrutinib as in Fig 3A. Western blot staining for downstream effectors pPRAS40 (T246), pP70S6K (T389) and pS6 (S235/236). **E. Combination of duvelisib with dexamethasone or ibrutinib leads to inhibition of cap-dependent translation**. DOHH2 (left) and SU-DHL-4 (right) treated as in Fig 3A and western blot stained for p4EBP1 (S65), c-MYC, peIF4E (s209) and cleaved PARP. The total eIF4E blots demonstrate that equal amounts of protein were loaded per lane.

To further our understanding of the synergy seen in combination testing, we examined the effect of duvelisib combinations on PI3K signaling in the sensitive cells lines using phosphorylation of the downstream PI3K effector, AKT (pAKT), as an indicator of PI3K pathway activity. Transformed follicular lymphoma DoHH2 cells were treated with duvelisib, dexamethasone, ibrutinib or the combination of duvelisib with dexamethasone or ibrutinib and cells were isolated for western blot analysis at 1 hour, 6 hour and 24 hours post-treatment (Fig 3A, top panel). While duvelisib efficiently inhibits pAKT (S473) at the early time points, AKT is re-phosphorylated by 24 hours (Fig 3A, top panel). Dexamethasone has no effect on pAKT alone, while ibrutinib slightly inhibited pAKT compared to DMSO (Fig 3A, top panel). However, the combination of duvelisib with dexamethasone more effectively inhibited pAKT at 24 hours compared to duvelisib alone (Fig 3A, top panel). Likewise, the combination of duvelisib and ibrutinib led to profound pAKT inhibition at 24 hours (Fig 3A, top panel). The drugs as single agents and in combination were also tested in the DLBCL SU-DHL-4 cell line, where the combination of duvelisib with either dexamethasone or ibrutinib lead to near complete inhibition of pAKT(S473) through the 24 hour time point (Fig 3A, bottom panel), superior to any of the single agents. These data suggest that the combination treatments prevent a rebound of PI3K signaling and this prolonged inhibition of the PI3K pathway enhances growth inhibition and can lead to cell death.

The reactivation of the PI3K pathway when cells are treated with duvelisib alone as measured by pAKT(S473) could be due to compensatory activation of other PI3K isoforms, or through activation of the mTOR pathway, which is known to phosphorylate AKT on Ser473 [20]. To address these possibilities, cells were treated with inhibitors that blocked different components of the PI3K and mTOR pathways, and the phosphorylation of AKT on Ser473 was assayed over a 24 hour period. The pan PI3K inhibitor, GDC-0941, was not able to inhibit the re-phosphorylation of pAKT (Ser473) at 24 hours (Fig 3B) indicating that this event was independent of the class I PI3K isoforms. Inhibition of ERK activity had no effect on pAKT (Fig 3B). The dual PI3K/mTOR inhibitor PF-04691502, however, prevented the re-phosphorylation of pAKT at 24 hours (Fig 3B), suggesting that mTOR activity is responsible for reactivation of AKT. To further refine whether this activity was related to mTORC1 or mTORC2, selective TORC inhibitors were utilized. Duvelisib and the mTORC1/2 dual inhibitor AZD-8055 were effective at inhibiting pAKT(Ser473) at 1 hour, while the mTORC1 inhibitor rapamycin was not active (Fig 3C, left panel), supporting the understanding that mTORC2 and PI3K activity can regulate the phosphorylation of pAKT(S473) [20–22]. To assess the role of mTOR in the setting of reactivation of pAKT after duvelisib treatment, cells were treated with duvelisib for 24 hours and then treated with either mTORC inhibitor or duvelisib for one additional hour. Retreatment with duvelisib or mTORC1 inhibitor rapamycin were unable to restore PI3K pathway inhibition, however, the addition of AZD-8055 inhibited pAKT (Fig 3C, right panel). These data indicate that mTOR inhibition can suppress re-phosphorylation of pAKT after duvelisib single agent treatment and that mTORC2 activity likely plays a key role in this process.

### Duvelisib combinations reduce activation of effectors downstream of mTORC1

While the activation of mTORC2 between 12–24 hours appeared to be responsible for the re-phosphorylation AKT at Ser473 after duvelisib treatment, we wished to determine if mTORC1-related activity was also concomitantly enhanced. To investigate this possibility, we evaluated activating phosphorylation of the mTORC1 signaling effectors PRAS40, P70S6K, and S6 in response to single agent duvelisib, dexamethasone, or ibrutinib and compared these effects to the combination of duvelisib and either dexamethasone or ibrutinib. Treatment of DoHH2 and SU-DHL-4 cell lines with duvelisib lead to inhibition of activated mTORC1 signaling effectors pPRAS40, pS6, and to a lesser extent, pP70S6K, at early timepoints (1 and 6hrs) (Fig 3D). However, after 24 hours, there is a general loss of this inhibition of activated downstream mTORC1 signaling effectors, particularly with respect to pPRAS40 (Fig 3D). Single agent dexamethasone and ibrutinib also show some inhibition of pPRAS40 and pS6 at early time points, particularly in the DoHH2 cell line, however, re-phosphorylation of these proteins generally occurs by 24 hours (Fig 3D). In contrast, duvelisib in combination with dexamethasone or ibrutinib effectively suppresses the re-phosphorylation of PRAS40, S6 and P70S6K at 24 hours. Additionally, the effect of the duvelisib combinations on cap-dependent translation was evaluated by measuring phosphorylation of 4EBP1. Duvelisib treatment of DoHH2 and SU-DHL-4 cell lines for 6 hours had a modest effect on inhibiting p4EBP1(S65) and peIF4E(S209), leading to a small decrease in the cap-dependent translation of c-MYC; whereas, single agent dexamethasone or ibrutinib had little effect (Fig 3E). In contrast, duvelisib in combination with dexamethasone had a profound effect on the inhibition of p4EBP1 and c-MYC translation in both cell lines (Fig 3E) and decreased pEIF4E in DoHH2. Importantly, these changes correlated with increases in cleaved PARP indicating a corresponding entry into apoptosis. These findings were less pronounced with the combination of duvelisib and ibrutinib, where a decrease in p4EBP1 and more minor decrease in c-MYC protein were seen in both cell lines (Fig 3E). These overall findings suggest that duvelisib in combination with either dexamethasone or ibrutinib may more effectively suppress mTORC1-mediated protein synthesis and cap-dependent translation in these aggressive lymphoma cell lines compared to monotherapy with these agents.

### Duvelisib combination therapy results in significantly greater tumor xenograft growth inhibition compared to monotherapies

**Fig 4:**
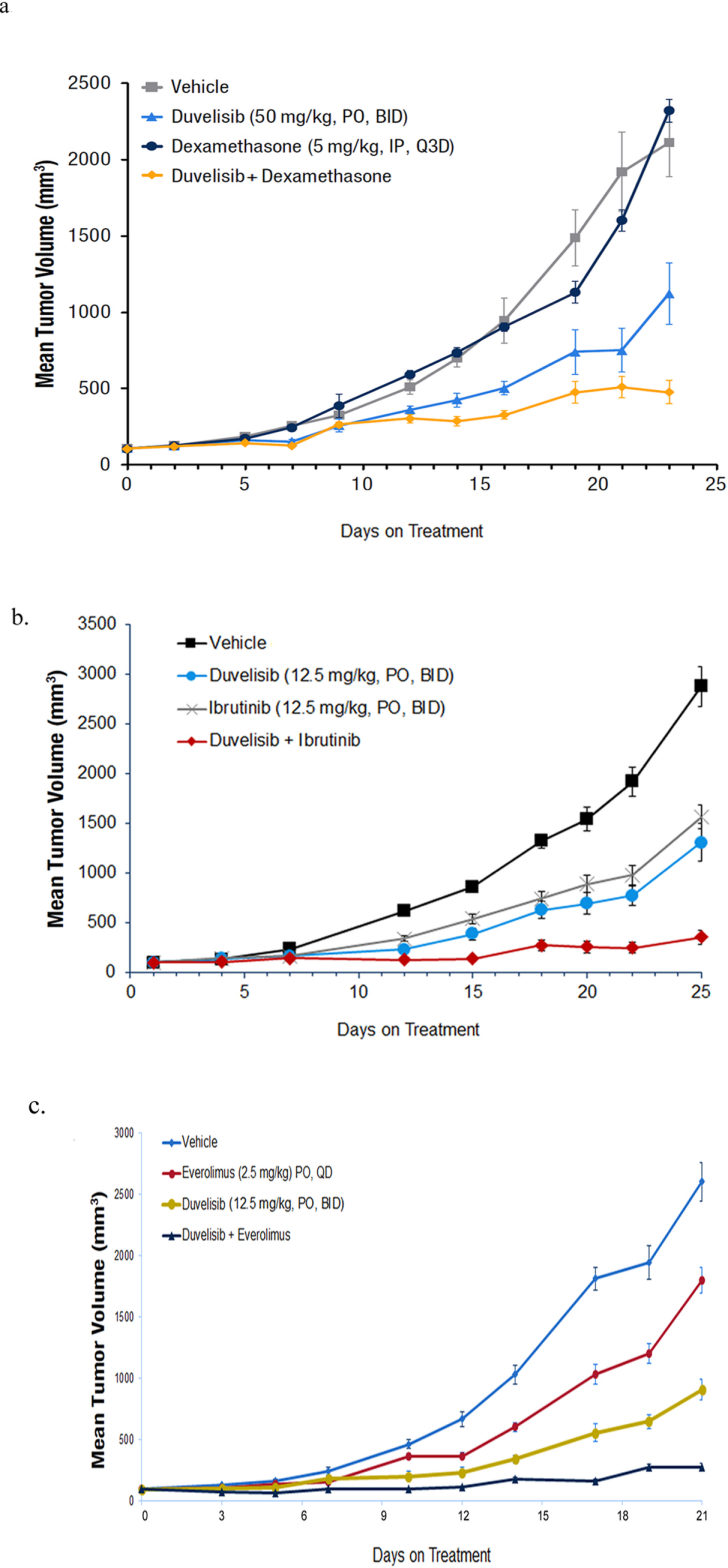
Duvelisib in combination with dexamethasone, ibrutinib or everolimus results in greater tumor growth inhibition compared to monotherapies. DOHH2 xenografts treated with duvelisib (50 mg/kg, PO, BID), dexamethasone (5 mg/kg, IP, Q3D) or the combination (**A**) or duvelisib (12.5 mg/kg, PO, BID), ibrutinib (12.5 mg/kg, PO, BID) or the combination (**B**). The combination groups showed significant growth inhibition compared to the monotherapies (**A**: p ≤ 0.006; **B**: p ≤ 0.001) **C**. SuDHL-4 xenografts treated with duvelisib (12.5 mg/kg PO, BID), everolimus (2.5 mg/kg, PO, QD) or the combination. Tumor growth was significantly inhibited in the combination group compared to monotherapies (p < 0.008).

To further validate the in vitro growth inhibition and mechanistic findings that were identified in the high throughput screen and PI3K/mTOR pathway studies, the effect of duvelisib, alone or in combination, was evaluated in an in vivo lymphoma xenograft model system. Based on the in vitro activity, it is expected that duvelisib in combination with dexamethasone, ibrutinib, or the mTOR inhibitor everolimus, would show enhanced tumor growth inhibitory activity compared to the effect of single agents. DoHH2 and SU-DHL-4 lymphoma xenograft studies confirmed this prediction, where duvelisib in combination with either dexamethasone, ibrutinib, or everolimus showed greater anti-tumor growth inhibitory activity compared to monotherapies (Fig 4A–4C). In all cases, tumor growth inhibition in the combination groups was significantly more effective than in the single agent groups. Although not curative in these fast-growing xenografts, the combinations of duvelisib and ibrutinib (Fig 4B) and duvelisib and everolimus (Fig 4C) led to a static growth rate and even regression in some tumor-bearing animals. This type of delayed or inhibited growth would correspond to a survival advantage.

## Discussion

Duvelisib is an orally available, potent dual inhibitor of PI3K-δ and PI3K-γ. In vitro studies, in the absence of a tumor microenvironment, demonstrated decreased viability of primary human neoplastic B CLL cells after duvelisib treatment [23,24]. These effects were modest, but demonstrate a role for intrinsic PI3K signaling in the survival of B-cell malignancies in the absence of specific microenvironmental signals [23,24]. As with many targeted agents, the clinical activity is considerably less in more aggressive lymphomas, including diffuse large B cell lymphoma [19]. We speculated that these aggressive lymphomas have aberrant signaling in multiple pathways that confer cell autonomous growth and are less dependent on survival cues from the microenvironment. This lack of dependence on external survival signals is typified by cell lines. Therefore, using a high throughput cell line screen in conjunction with duvelisib, we sought to identify agents that target complementary pathways to identify potentially more effective combination regimens. We first determined the single agent activity of duvelisib against the panel of cell lines (Fig 1A and 1B and Table 1). Fourteen of 20 cell lines tested showed at least 50% maximal growth inhibition in response to three days of duvelisib exposure with a median GI50 of 0.59 μM. The greatest sensitivity was seen in the DLBCL or transformed FL cell lines, with less activity seen in MCL, MM or T-cell lymphoma lines. The one exception was a cutaneous T-cell lymphoma cell line, HH, which did show considerable sensitivity (GI50 = 10 nM). There was no consistent trend in sensitivity among activated B cell (ABC) vs. germinal center B cell (GCB) DLBCL cell lines, but all three transformed FL cell lines tested were very sensitive to duvelisib.

To identify agents that would enhance the activity of duvelisib in these same cell lines, a high-throughput combination screen was carried out with 30 agents that are relevant to the treatment of hematological malignancies (Fig 2A). This included the use of standard-of-care drugs to treat lymphoid lymphomas (e.g. dexamethasone, doxorubicin, vinblastine, chlorambucil and bendamustine), emerging drugs for the treatment of lymphoid lymphomas (e.g. venteoclax (ABT-199), bortezomib, carfilzomib, romidepsin, and lenalidomide) or drugs that target proteins involved in B-cell receptor signaling including SYK (PRT062607), BTK (ibrutinib, spebrutinib), PI3K/AKT/mTOR (everolimus, AZD8055, GDC-0941, MK-2206 and perifosine), PKCbeta (enzastaurin) and NF-kB (Bay 11–7082 and BMS345541). Combination activity of duvelisib with dexamethasone was considerably higher than with other standard of care agents, while weak combination activity with chlorambucil and doxorubicin was noted (Fig 2A and 2B). Other DNA damaging agents such as bendamustine and vinblastine showed less pronounced effects, a result that might be attributable to the fact that multiple rounds of DNA division are required for cytostatic or cytotoxic activities to fully manifest. Overall, the combination activities observed with standard-of-care drugs provide encouragement that duvelisib will provide benefit to patients with B cell malignancies when paired with components of the combination drug regimen CHOP (Cyclophosphamide, Doxorubicin, Vincristine and Prednisolone).

Strong combination activity and good breadth-of-activity was observed for many of the emerging drugs in the screen (Fig 2A and 2B). Combination activity with venetoclax (ABT-199) was most pronounced in DLBCL and transformed FL cell lines, but was quite variable among these lines. There was no apparent association of sensitivity with either ABC or GCB lymphoma subtypes or the presence or absence of a BCL2 translocation. Importantly, the screen contained two proteasome inhibitors (bortezomib and carfilzomib) and two BTK inhibitors (ibrutinib and spebrutinib), which showed similar synergy patterns. Having multiple compounds that affect the same target(s)/pathway is important when performing large screens, as shared patterns of combination activity across the cell line panel help to validate activities and can highlight the importance of cell line genotypes or specific network contexts for duvelisib-induced combination activities.

To better understand the mechanism of the strong synergy observed with duvelisib in combination with dexamethasone or ibrutinib, we undertook an investigation of the PI3K/AKT/mTOR signaling pathways after single agent treatment or treatment with duvelisib in combination with these agents. Continuous treatment with duvelisib resulted in initial suppression of pAKT that returned between 12–24 hours. Analysis with various inhibitors of the PI3K or mTOR pathway revealed that the re-activation of AKT after duvelisib treatment was dependent on mTOR activity and more specifically, on the activity of mTORC2. In contrast, the combination of duvelisib with either dexamethasone or ibrutinib prevents mTORC2 activation, leading to inhibition of downstream mTOR signaling. This includes the inhibition of the mTORC1 signaling effectors pPRAS40, pP70S6K, and pS6, as well as the inhibition of cap dependent translation initiation via prevention of p4EBP1 phosphorylation, leading to the loss of c-MYC. Importantly, the suppression of downstream mTOR signaling that is observed with the combination treatments correlates with enhanced growth inhibition and induction of cleaved PARP. In addition, significant in vivo activity is seen with duvelisib in combination with not only dexamethasone and ibrutinib but also in combination with the mTOR inhibitor everolimus. Interestingly, in vitro synergy was also noted in the high-throughput screen with the mTOR inhibitors everolimus and AZD-8055. These in vitro and vivo findings not only support the signaling data but confirm the importance of suppressing the mTOR pathway in order to obtain maximal benefit from duvelisib-mediated PI3K-δ and PI3K-γ inhibition [25].

The results of this high-throughput synergy screen with duvelisib in multiple lymphoma/myeloma cell lines show multiple agents that show significant combination activity both in vitro and in vivo. This synergy was particularly striking in lymphoma cell lines treated with duvelisib in combination with either ibrutinib or dexamethasone. Detailed biochemical analysis revealed these combinations acted to suppress mTOR-induced reactivation of AKT after long term duvelisib exposure, preventing both mTORC1 and mTORC2 mediated survival and growth signals. Overall, these studies suggest a scientific rationale for combining duvelisib with a variety of agents in clinical trials targeting aggressive lymphomas, and, in particular, point to a strategy that includes agents that suppress mTOR signaling, such as dexamethasone, ibrutinib or everolimus.

## Materials and methods

### Ethics statement

This study was carried out in strict accordance with the recommendations in the Guide for the Care and Use of Laboratory Animals printed by the National Research Council of the National Academies. The protocol was approved by the Institutional Animal Care and Use Committee of Infinity Pharmaceuticals, Inc. Every effort was made to minimize animal suffering through the entirety of the study.

### High throughput combination screen

A high throughput combination screen evaluating duvelisib alone and in combination with a panel of 35 diverse compounds was conducted by Horizon Discovery (Cambridge, MA). These compounds were tested in 20 cell lines provided by Horizon Discovery (Cambridge, MA) including diffuse large B-cell (DLBCL), follicular, T-cell, and mantle cell lymphomas, and multiple myeloma. Growth inhibition (GI) was measured after 72 hours by ATPLite (Perkin Elmer, Waltham MA) in a 6x6 or 9x9 dose combination matrix. Combination effects in excess of Loewe additivity were measured using Chalice Software (Horizon Discovery, Cambridge, MA) and expressed as a scalar measure (synergy score) to characterize the strength of the synergistic interaction.

### Cell cultures

DOHH2 cells were obtained from the German Collection of Microorganisms and Cell Cultures (Leibniz-Institut DSMZ, Braunschweig, Germany). SuDHL-4 cells were obtained from the American Type Culture Collection (ATCC, Manassas, VA). Both cell lines were maintained in Roswell Park Memorial Institute medium-1640 (RPMI-1640) (Life Technologies, Grand Island, NY) supplemented with 10% heat-inactivated fetal bovine serum (FBS) (Sigma-Aldrich, St. Louis, MO) and 100 units/ml penicillin and 100ug/ml streptomycin (Life Technologies, Grand Island, NY). All cell cultures were maintained at 37°C in a humidified atmosphere of 5% CO_2_.

### Compounds

Duvelisib (IPI-145) was prepared in Infinity Laboratories using methods described in Winkler et al [16]. Dexamethasone (D2915) was purchased from Sigma- Aldrich, (St. Louis, MO) and all other compounds were purchased from Selleck Chemicals (Houston, TX): Ibrutinib (S2680), Prednisone (S1622), MK-2206 (S1078), SCH 772984 (S7101),GDC-0941 (S1065), PF-04691502 (S2743), Rapamycin (S1039), AZD-8055 (S1555) and Venetoclax (S8048).

### Subcutaneous xenograft models

DOHH2 cells (2x10^6^ cells per mouse in 0.1ml of sterile PBS) and SuDHL-4 cells (5x10^6^ cells per mouse in 0.1ml of sterile PBS) were implanted subcutaneously into the right flank of 5–6 week old CB17.SCID female mice (Taconic, Hudson, NY). Mice were housed 4 per cage and offered food and water ad libitum. Environmental controls for the animal room were set to maintain 18 to 26°C, a relative humidity of 30 to 70%, a minimum of 10 room air changes/hour, and a 12-hour light/12-hour dark cycle.

To determine the effect various compounds had on tumor growth in vivo, mice were randomized into treatment groups (N = 12–15 per group) when tumors reached an average volume of 100 mm^3^ in volume. All compounds except dexamethasone were dosed orally either once (QD) or twice daily (BID) as indicated in the figure legends. Dexamethasone (002459) for in vivo use was purchased from Henry Schein, (Dublin, Ohio). Dexamethasone was dosed intraperitoneally (IP), every third day (Q3D). Compounds were formulated as follows: duvelisib in 5% NMP, 95%PEG400; dexamethasone was diluted in 0.9% sodium chloride saline; ibrutinib in 20% HPβCD; everolimus in 6.25% Ethanol, 5% PEG400, 5% Tween80.

The selected compound doses for the in vivo xenograft studies were chosen to maximize combination effects. Based on in vivo tolerability studies (data not shown) the maximum tolerated dose for duvelisib was shown to be 50mg/kg BID. All drug combinations were well tolerated for the duration of the study.

Body weights and tumor caliper measurements (length and width) were recorded twice weekly for the duration of the study. Tumor volumes ((length x width^2^)/2) never exceeded our IACUC approved tumor volume threshold of 3000 mm^3^. No animals suffered adverse outcomes or died while on study. Body weights never declined more than our IACUC approved maximum weight loss threshold of 20%. At the end of the study all mice were humanely euthanized by trained pharmacologists using inhaled CO_2._

### Western blot analysis

Protein lysates were prepared using RIPA lysis buffer (Sigma-Aldrich, ST. Louis, MO) with the addition of the complete EDTA-Free Protease Inhibitor Cocktail (Roche, Mannheim, Germany) and the HALT Phosphatase Inhibitor Cocktail (Thermo Fisher Scientific, Waltham, MA). Equivalent amounts of protein were loaded per lane, separated by electrophoresis using 4–12% Bis-Tris gels (BIORAD, Hercules, CA) and transferred onto PVDF membranes (Invitrogen, Carlsbad, CA). Membranes were probed with the following primary antibodies: pAKT S473 (Cell Signaling Technologies, Beverly, MA) (CST-4060), pPRAS40 T246 (CST-2997),pP70S6K T389 (CST-9205), pS6RB S235/236 (CST-4858), p4EBP1 S65 (CST-13443),c-MYC (CST-13987), pEIF4E S209 (CST-9741), Total EIF4E (CST-9742), Cleaved PARP (CST-9546), Beta-Actin (Santa-Cruz Biotechnology, Dallas, TX) (SC-47778). After secondary antibody incubations, proteins were detected on film with the ECL chemiluminescent detection reagent (GE Healthcare Life Sciences, Pittsburg). The beta actin blots corresponding to Fig 3A–3D can be found in S1 Fig and demonstrate that equal amounts of protein were loaded per lane.

## Supporting information

S1 Fig Beta actin western blots corresponding to Fig 3A–3D. (TIF)

S1 Text Arrive guidelines checklist. (DOCX)
