# Supplementary figures and images for: The phosphoinositide-3 kinase (PI3K)-δ,γ inhibitor, duvelisib shows preclinical synergy with multiple targeted therapies in hematologic malignancies
Source: PLoS One. 2018 Aug 1;13(8):e0200725. doi: 10.1371/journal.pone.0200725 (PMC6070190; doi:10.1371/journal.pone.0200725)

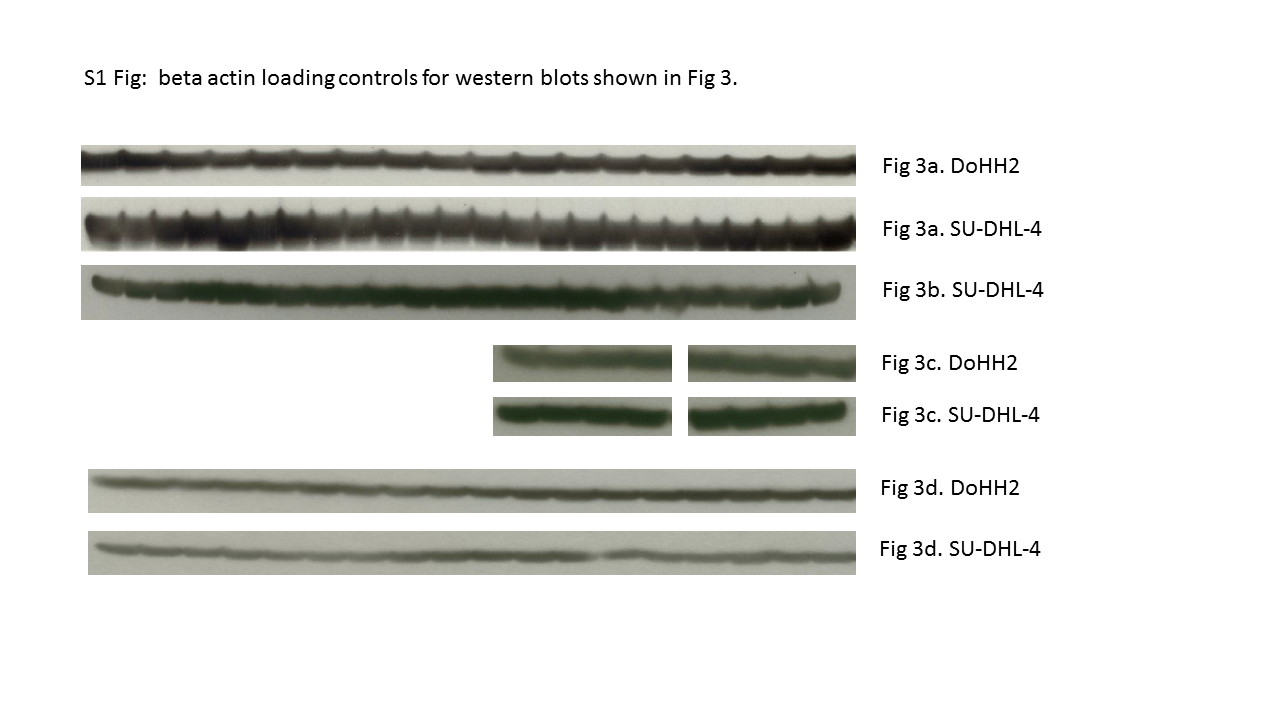

Supplement: S1 Fig — (TIF) [file pone.0200725.s001.tif]
