# Supplementary material for: The phosphoinositide-3 kinase (PI3K)-δ,γ inhibitor, duvelisib shows preclinical synergy with multiple targeted therapies in hematologic malignancies
Source: PLoS One. 2018 Aug 1;13(8):e0200725. doi: 10.1371/journal.pone.0200725 (PMC6070190; doi:10.1371/journal.pone.0200725)

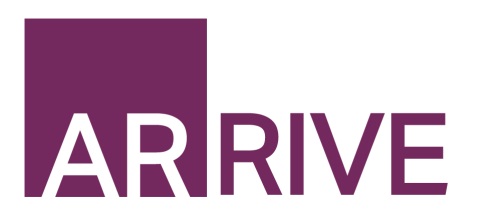


The ARRIVE Guidelines Checklist

Animal Research: Reporting In Vivo Experiments

Carol Kilkenny^1^, William J Browne^2^, Innes C Cuthill^3^, Michael Emerson^4^ and Douglas G Altman^5^

*^1^The National Centre for the Replacement, Refinement and Reduction of Animals in Research, London, UK, ^2^School of Veterinary Science, University of Bristol, Bristol, UK, ^3^School of Biological Sciences, University of Bristol, Bristol, UK, ^4^National Heart and Lung Institute, Imperial College London, UK, ^5^Centre for Statistics in Medicine, University of Oxford, Oxford, UK.*

|  | | ITEM | RECOMMENDATION | Section/ Paragraph |
| --- | --- | --- | --- | --- |
| 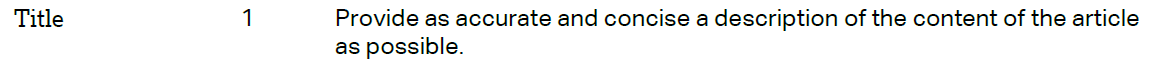 | | | Title |  |
| 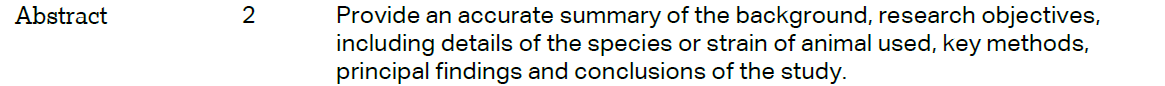 | | | Abstract |  |
| INTRODUCTION | | |  |  |
| 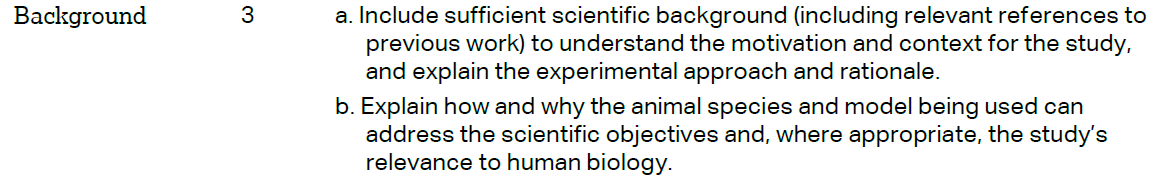 | | | Introduction |  |
| 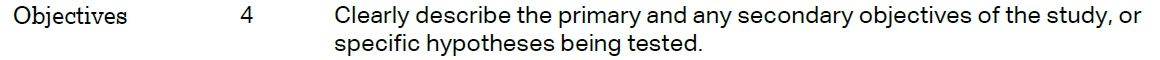 | | | Introduction lines 61-69 |  |
| METHODS | | |  |  |
| 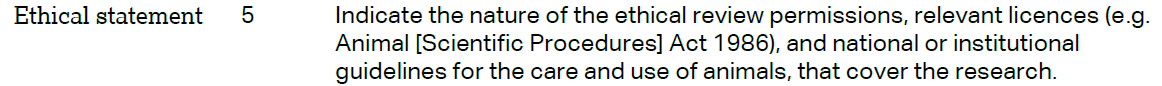 | | | Ethics Statement lines 298-303 |  |
| 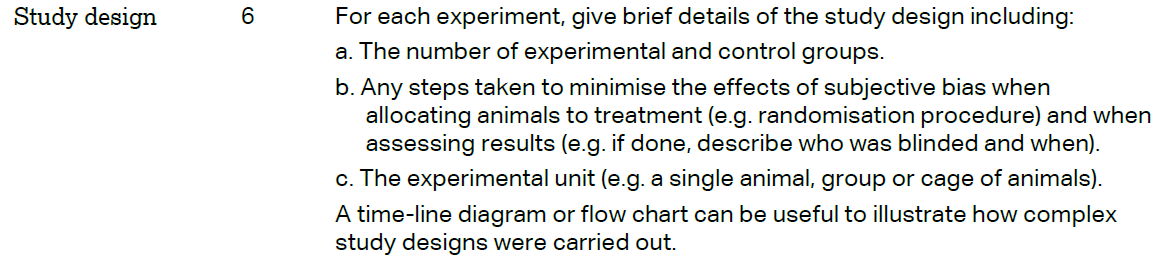 | | | Results and Materials and Methods |  |
| 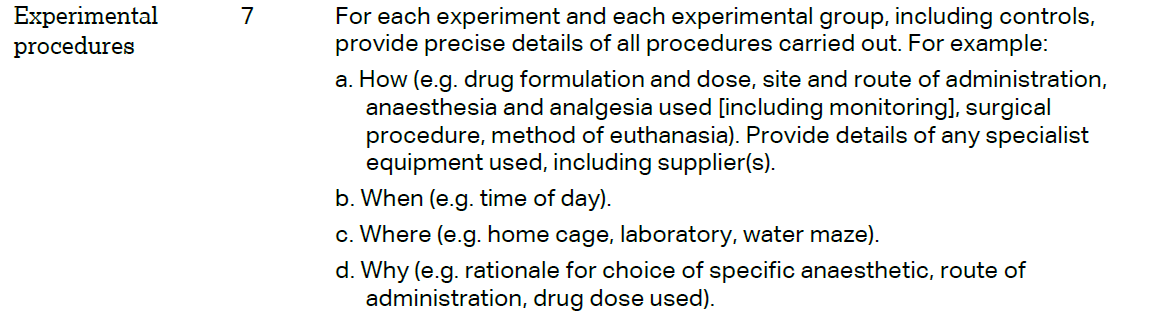 | | | Material and Methods |  |
| 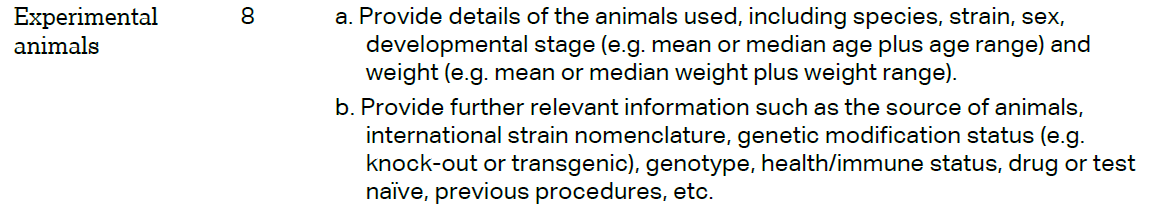 | | | Material and Methods subsection subcutaneous xenograft models. |  |

The ARRIVE guidelines. Originally published in *PLoS Biology*, June 2010^1^

| 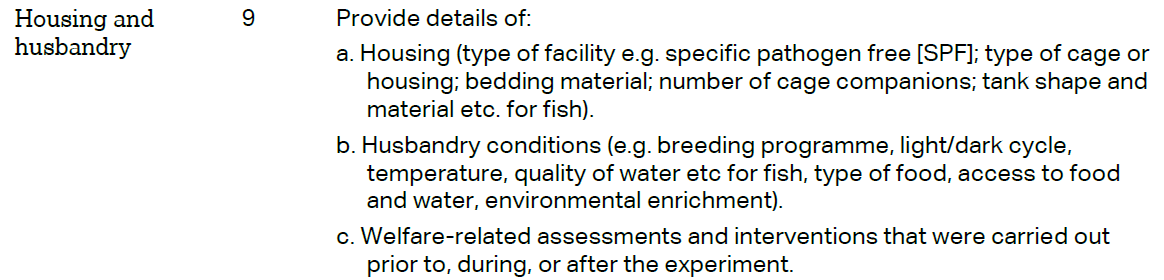 | Material and Methods subsection subcutaneous xenograft models. | |
| --- | --- | --- |
| 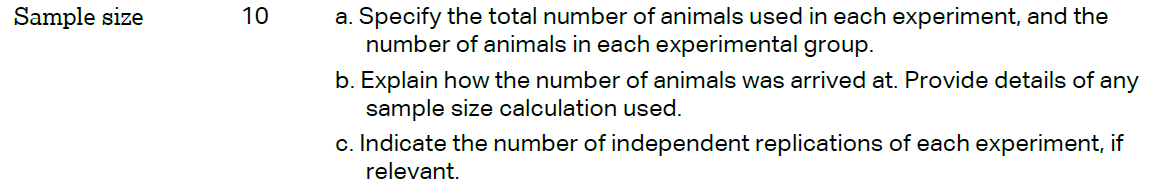 | Material and Methods subsection subcutaneous xenograft models. | |
| 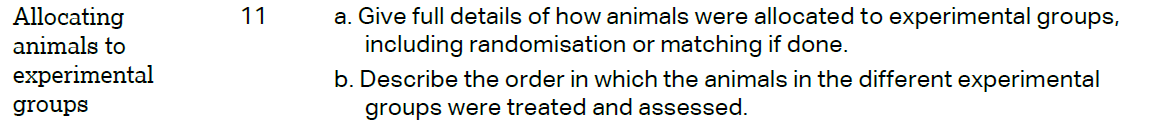 | Material and Methods subsection subcutaneous xenograft models. | |
| 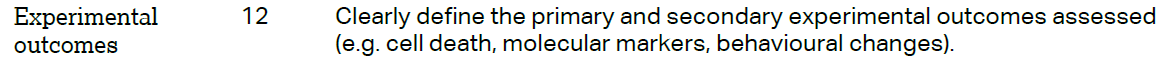 | Material and Methods | |
| 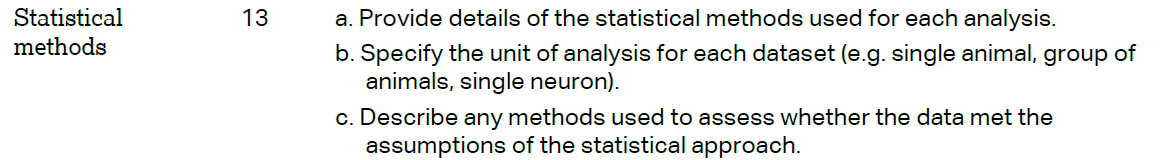 | Material and Methods | |
| RESULTS |  | |
| 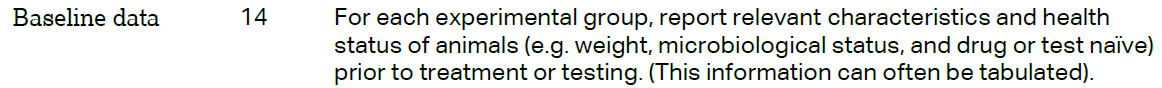 | Material and Methods subsection subcutaneous xenograft models. | |
| 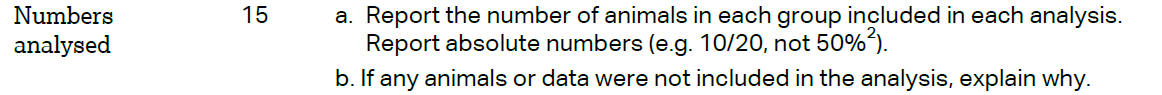 | Material and Methods subsection subcutaneous xenograft models. | |
| 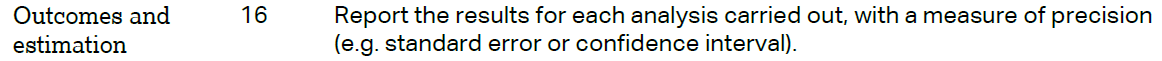 | Results and Figures | |
| 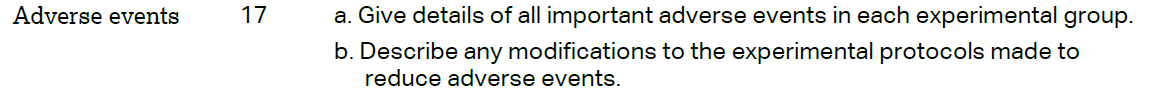 | Material and Methods subsection subcutaneous xenograft models. | |
| DISCUSSION |  | |
| 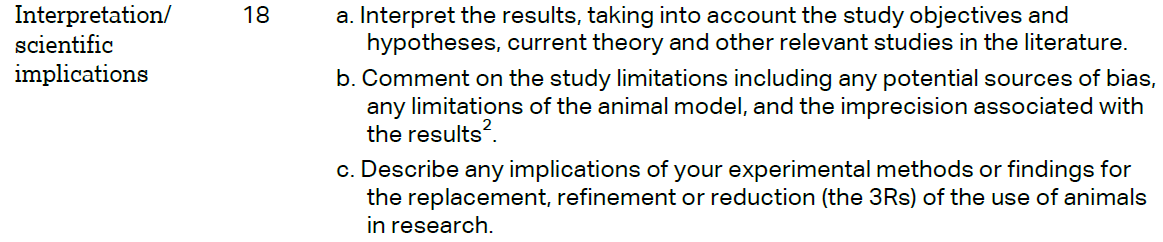 | Discussion | |
| 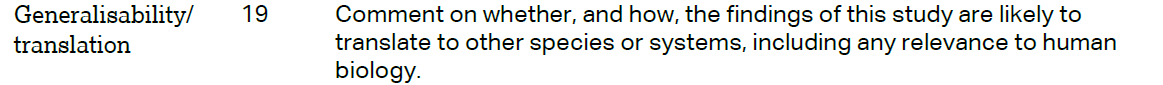 | Discussion lines 281-291 | |
| 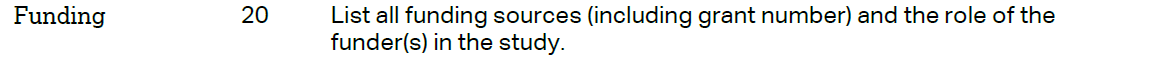 | | Entered as required during submission. All funding was provided by Infinity Pharmaceuticals, Inc. |


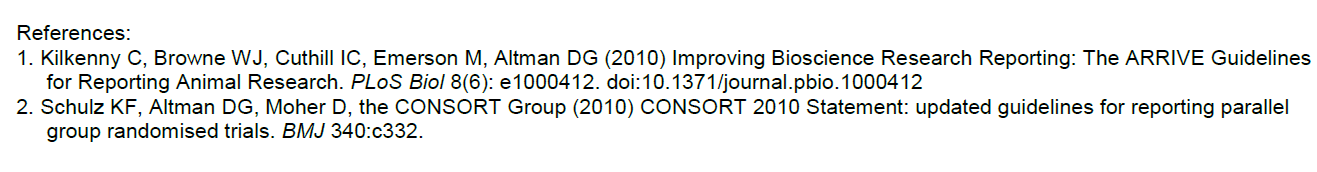

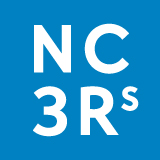

Supplement: S1 Text — (DOCX) [file pone.0200725.s002.docx]
